# Knowledge of cardiovascular diseases among university students in Egypt

**DOI:** 10.1038/s41598-025-34137-6

**Published:** 2026-01-21

**Authors:** Maha Ibrahim Adel, Mohamed Ashraf Hall, Inas Karawia

**Affiliations:** 1 Dental Public Health and Preventive Dentistry Department, Faculty of Dentistry, Pharos University in Alexandria, Alexandria, Egypt; 2 Alexandria Dental Research Center, Ministry of Health and Population, Alexandria, Egypt

**Keywords:** Knowledge, Awareness, Cardiovascular diseases, Students, Egypt, Cardiology, Diseases, Health care, Medical research, Risk factors

## Abstract

Cardiovascular diseases (CVDs) are the leading cause of death worldwide. Promoting early awareness and understanding of modifiable risk factors among young people is crucial for prevention. Yet, little is known about the level of awareness among university students in Egypt. This study aimed to evaluate university students’ knowledge of cardiovascular diseases (CVDs) and their associated risk factors, with a focus on general awareness, scientific understanding, and behavioral responses related to cardiovascular health. A cross-sectional analytical study was carried out among 300 university students using a pre-validated English questionnaire administered through Google forms. Participants were recruited from seven faculties at Pharos University. The questionnaire evaluated students’ knowledge of CVD risk factors and their overall awareness of cardiovascular conditions. The study revealed significantly higher knowledge scores among female participants (15.3 ± 2.6) compared to males (13.8 ± 4.0), (*p* = 0.007). The Faculty of Dentistry had the highest mean knowledge score (15.6 ± 3.1), with a significant decline in knowledge observed among students from applied sciences and engineering/arts (*p* = 0.04 and 0.002, respectively). While no significant differences were found between dentistry, pharmacy, or physical education students. Higher academic grades generally had better CVD knowledge compared to first-year students (12.6 ± 3.7), (*p* = 0.04). Furthermore, high awareness was noted in several areas, including the role of blood pressure control (87.6%), physical activity (85.1%), obesity (85.6%), smoking (90%), and aging (89.6%) in CVD risk. Moderate awareness was observed for family history (75.1%), blood sugar control (70.1%), and myocardial oxygen supply (70.6%). However, low awareness was noted regarding the role of HDL cholesterol (45.8%) and the effect of fatty foods on cholesterol (23.9%). Although university students showed a generally good awareness of major cardiovascular risk factors, notable knowledge gaps persisted—especially regarding dietary fats and cholesterol types. These results highlight the importance of implementing targeted educational programs to improve cardiovascular health awareness among young adults in Egypt.

## Introduction

Cardiovascular diseases (CVDs), which include a range of heart and blood vessel disorders, are the leading cause of death globally. Each year, 17.9 millions of people die from CVD, accounting for about 31% of all deaths worldwide^1^. These conditions include coronary, cerebrovascular, and peripheral arterial diseases, as well as rheumatic, congenital heart diseases, deep vein thrombosis, and pulmonary embolism^2^.

Over the past two decades, young people in developed countries have shown a high prevalence of cardiovascular risk factors, including obesity, inactivity, and poor diet^3^. Other identified risks include smoking, hypertension, and diabetes, while non-modifiable factors include age, gender, rheumatic heart disease, and family history^4^. According to the WHO (2021), the Eastern Mediterranean Region shows high rates of CVD risk factors, with hypertension affecting 28–41% and hypercholesterolemia 14–52% of the population. These conditions can cause acute events like heart attacks and strokes, mainly due to fatty deposits blocking blood flow to the heart or brain^5^.

Egypt faces a concerning increase in coronary artery disease (CAD) cases and deaths^6,7^. Early development of CAD risk factors is closely linked to lasting atherosclerotic changes and predicts future disease risk^8^. According to the 2018 Ministry of Health report, approximately 300,000 Egyptians aged 5–15 were affected by rheumatic heart disease (RHD)^8,9^, with Egypt recording the region’s highest number of RHD-related deaths (9,168)^10^.

Early development of major cardiovascular risk factors is strongly linked to clustered risks and unhealthy behaviors^11^. Conditions like hypertension, dyslipidemia, glucose intolerance, and overweight may be tolerated in youth^12^, but when combined with poor diet, smoking, or inactivity, they increase morbidity and mortality from cardiovascular disease later in life^13^. Early exposure to these risks can cause lasting arterial changes, leading to atherosclerosis^14–16^.

Despite the growing burden of cardiovascular diseases (CVDs) in Egypt, limited research has explored the level of knowledge and awareness of CVDs and their modifiable risk factors among young adults, particularly university students. This gap is critical, as early understanding of risk factors and warning signs can influence lifelong health behaviors and improve disease outcomes^16,17^. , This study aimed to assess the level of knowledge about cardiovascular diseases and their risk factors among students at a private university in Egypt. emphasizing general awareness, scientific knowledge, and behaviors influencing cardiovascular health.

## Materials and methods

### Study design and setting

This study was a cross-sectional analytical investigation conducted among students of different faculties at Pharos University in Alexandria, Egypt, during the second semester of the 2024–2025 academic year. The research was carried out following ethical approval from the university’s ethics committee, under registration number (UREAC-02-3-376) approved on 22-3-2025. Participants were provided with a cover letter at the start of the Google Form, outlining the study’s objectives and confirming its voluntary and anonymous nature. The estimated time to complete the form was also included. Written informed consent was obtained from all participants before they filled out the questionnaire. The study adhered to the STROBE guidelines for observational studies^18^, ensuring methodological rigor and transparency. All methods were conducted in accordance with the Declaration of Helsinki and relevant institutional regulations.

### Sample size and sampling technique

The sample size was calculated using Epi-Info software, guided by data from a previous study that showed 74% of undergraduate students were aware that managing blood pressure can help reduce the risk of heart disease^19^. Using a 95% confidence interval, an alpha error of 0.05, and a power of 0.80, the estimated sample size was 296, which was rounded up to 300 students for convenience.

Participation in the study was entirely voluntary. The inclusion criteria were undergraduate students from the selected faculties at Pharos University who gave their consent to participate. Students who had already graduated (intern students) or refused to complete the questionnaire were excluded.

A convenience sampling technique was used to recruit undergraduate students from six faculties at Pharos University: Dentistry, Pharmacy, Applied Health Sciences, Physical Therapy, Engineering, and Art and Design. Students from all academic years (first through fifth) were invited to take part.

Recruitment was carried out through two approaches:

- Online distribution of the questionnaire link via university email groups and student platforms.
- Face-to-face recruitment on campus to ensure adequate representation from all faculties.

Data collection continued until the target sample size of 300 completed responses was reached. Because all items in the Google Form were mandatory, the system did not allow submission unless all questions were answered; therefore, there were no missing data or partially completed forms.

To ensure privacy and encourage honest responses, the questionnaire was anonymous and confidential. Informed written consent was obtained from each participant before completing the survey.

### Data collection tool

A previous pre-validated questionnaire^19^ in English was used. The tool was adopted exactly as it was published without any modifications or rewording. Its validity and reliability were established by the original authors, who reported a Cronbach’s alpha of 0.82. Since the current study used the same instrument in its original form, no additional pilot testing or reliability assessment was required.

The distributed Google Form questionnaire contained two sections: the first section was demographic data, asking about name, age, faculty, sex, and Academic level. The second section comprises 20 questions, divided into three main categories. Thirteen items assess participants’ knowledge of cardiovascular disease, including its common forms and key risk factors—both modifiable (such as smoking, obesity, hypertension, and physical inactivity) and non-modifiable (such as age and family history). This category also addresses preventive measures aimed at reducing CVD risk through lifestyle choices and medical management. Five questions focus on more advanced scientific concepts related to cardiovascular physiology, including how factors like blood sugar levels affect heart function and the role of coronary blood flow in oxygen delivery to the myocardium. This section also tests understanding of common misconceptions, such as those involving HDL and LDL cholesterol or the urgency required in treating acute cardiac events. The remaining two questions explore behavioral responses and attitudes, particularly in emergency scenarios, assessing the respondents’ readiness to seek medical care and their awareness of how psychological stress may impact disease progression.

The questionnaire needed 5 to 10 min to be completed.

As online responses were insufficient to fulfill the required sample size, the remaining participants were surveyed in person on campus.

Each right answer was scored 1, the wrong answer was scored 0.

The total knowledge was evaluated as follows:

- Low knowledge: 0–7.
- Moderate knowledge: 8–14.
- High knowledge: 15–20.

These intervals were calculated by dividing the total number of items (20) into three equal categories to allow for a balanced comparison between low, moderate, and high knowledge levels. No external cut-off points were used.

### Statistical analysis

Data were coded and analyzed using the Statistical Package for the Social Sciences (SPSS, version 25.0; IBM Corp., Armonk, NY, USA). Qualitative variables were summarized as frequencies and percentages, while quantitative data (knowledge scores) were expressed as means and standard deviations. Independent sample *t*-tests and one-way ANOVA were used to compare mean knowledge scores across demographic and academic groups. Variables that showed significant associations in bivariate analyses were entered into a multiple linear regression model to identify independent predictors of cardiovascular health knowledge, adjusting for potential confounders such as sex, faculty, and academic year. Regression results were reported using β coefficients, 95% confidence intervals. No missing data were encountered, as all questionnaire items were mandatory in the Google Form, preventing incomplete submissions. the significance threshold was set at *p* < 0.05 to reduce the likelihood of Type I error.

## Results

Table 1 presents the demographic characteristics of the participants. The sample included 300 students, almost equally divided by sex, and represented a relatively young and homogeneous age group. Most participants were from health-related faculties, particularly Dentistry and Pharmacy, while fewer students were enrolled in non-health disciplines such as Engineering and Arts. Senior students (fourth and fifth years) represented the largest proportion of the sample.

**Table 1:** Distribution of the participants by demographic data.

	No.	%
Sex		
Male	155	51.7
Female	145	48.3
Mean age	21.35 ± 1.4	
Faculty		
Dentistry	110	36.8
Pharmacy	69	22.9
Applied	55	18.4
Physical	36	11.9
Eng/Art	30	10
Grade		
Fifth	81	40.3
Fourth	49	24.4
Third	36	17.9
Second	23	11.4
First	11	5.5

Table 2 presents the results of the linear regression analysis examining the relationship between demographic factors and knowledge of cardiovascular diseases (CVD). Female students demonstrated significantly higher knowledge scores than males. Students from Dentistry achieved the highest knowledge levels, while those from non-health faculties, including Applied Sciences and Engineering/Arts, exhibited significantly lower scores. Knowledge also tended to improve with academic grade, with fourth- and fifth-year students showing better understanding than lower-grade students.

**Table 2:** Relationship between participants’ demographic factors and the knowledge of CVD.

	Mean ± SD	B	t	Sig.	95%CI
Sex					
Male^Ref^	13.8 ± 4.0				
Female	15.3 ± 2.6	1.029	2.7	0.007*	[0.30–1.77]
Faculty					
Dentistry^Ref^	15.6 ± 3.1				
Pharmacy	14.4 ± 3.5	-0.49	-0.74	0.46	[–1.79–0.81]
Applied	14.3 ± 2.4	-1.62	-2.09	0.04*	[–3.18 - − 0.06]
Physical	14.4 ± 3.7	-0.71	-0.88	0.38	[–2.31–0.89]
Eng/Art	9.7 ± 3.9	-2.77	-3.14	0.002*	[–4.49 - − 1.05]
Grade					
First^Ref^	12.6 ± 3.7				
Second	13.1 ± 2.9	0.06	0.09	0.93	[–1.55–1.43]
Third	14.9 ± 3.2	0.16	0.23	0.81	[–1.58–1.26]
Fourth	14.8 ± 3.3	0.064	2.07	0.04*	[0.01–0.47]
Fifth	14.9 ± 3.4	0.157	2.11	0.04*	[0.01–0.31]

F = 4.23 *P* = 0.000 Adjusted R-square = 13.3%.

*Significance at p-value ≤ 0.05.

^Ref^the reference variable.

**Fig. 1:**
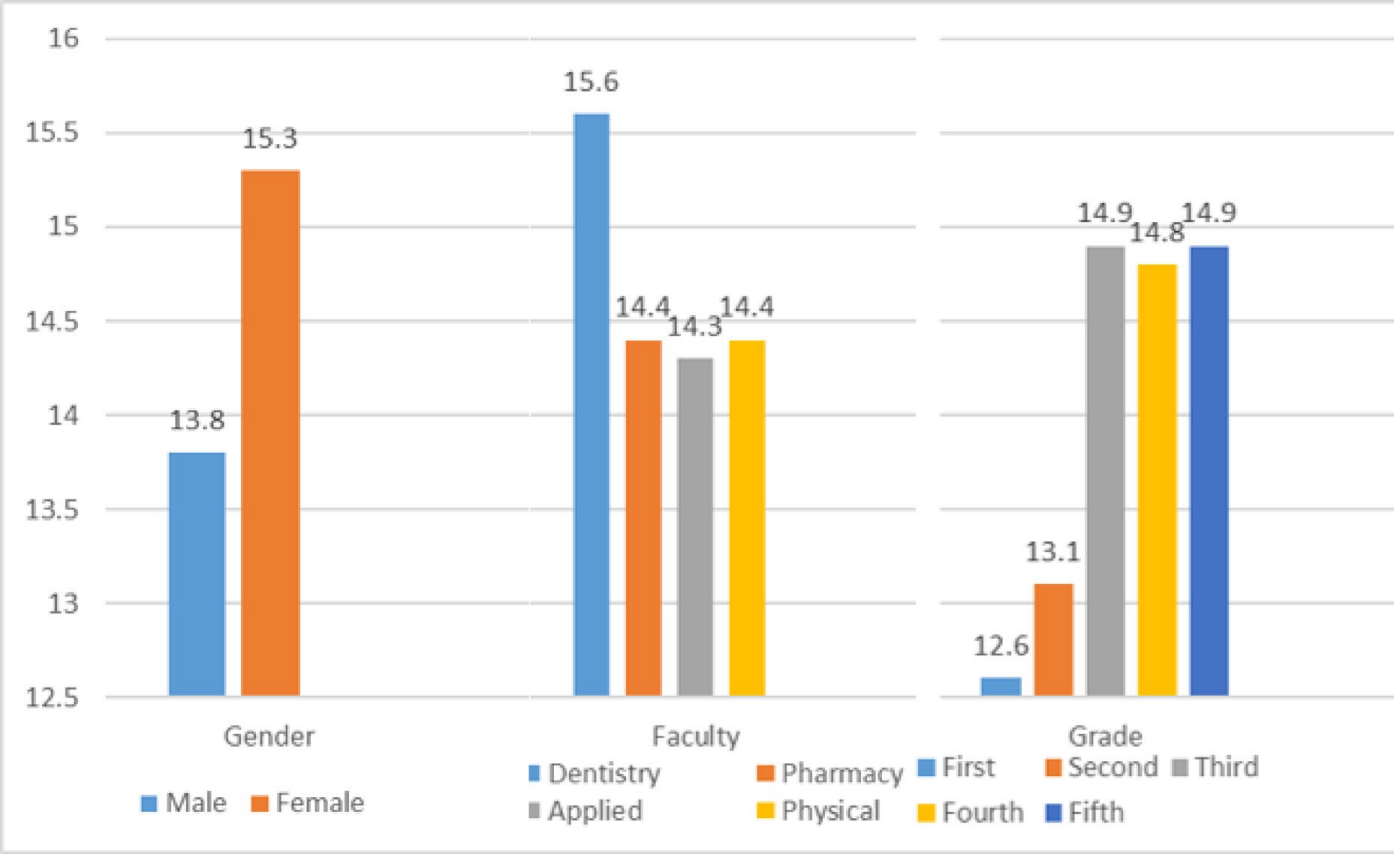
Comparison of mean knowledge scores among students according to gender, faculty, and academic grade.

As shown in Fig. 1, females demonstrated a high level of knowledge (15.3), whereas males showed a moderate level (13.8). Among faculties, Dentistry students exhibited the highest knowledge level (15.6), followed by Pharmacy, Applied Health Sciences, and Physical Therapy students, who all showed moderate levels (14.3–14.4). Regarding academic grade, first- and second-year students displayed moderate knowledge (12.6 and 13.1), while third-, fourth-, and fifth-year students reached the high category (14.8–14.9).

Table 3 illustrates participants’ awareness of major cardiovascular risk factors and preventive measures. Overall, students displayed high awareness regarding the harmful effects of smoking, the importance of controlling blood pressure, and the influence of obesity and aging on heart disease. However, knowledge was limited concerning the role of HDL cholesterol and dietary fat intake. Moderate levels of awareness were observed for items related to blood sugar control and the urgency of medical care in acute myocardial infarction. These results indicate that while general awareness of CVD is satisfactory, specific misconceptions about lipid profiles and diet persist and warrant targeted educational interventions.

**Table 3:** Awareness of risk factors and preventive measures for coronary heart disease among participants.

Statements	Right answer no (%)	Wrong answer no (%)
If you have a family history of coronary heart disease, you are at risk for developing heart disease	226 (75.1%)	74 (24.9%)
Keeping blood pressure under control will reduce a person’s risk for developing heart disease	263 (87.6%)	37 (12.4%)
If your “good” cholesterol (HDL) is high, you are at risk for heart disease	137 (45.8%)	163 (54.2%)
A person who has diabetes can reduce their risk of developing coronary heart disease if they keep their blood sugar levels under control	210 (70.1%)	90 (29.9%)
Regular physical activity will lower a person’s chance of getting heart disease	255 (85.1%)	45 (14.9%)
Do you think obesity influences the progression of coronary heart disease?	257 (85.6%)	43 (14.4%)
Coronary heart disease is a type of cardiovascular disease	257 (85.6%)	43 (14.4%)
Eating fatty foods does not affect blood cholesterol levels	72 (23.9%)	228 (76.1%)
Do you think smoking habits influence the progression of coronary heart disease?	270 (90.0%)	30 (10.0%)
Time is of no importance when it comes to treatment results for Acute Myocardial Infarction	90 (29.9%)	210 (70.1%)
Deep vein thrombosis and pulmonary embolism are a types of cardiovascular diseases	218 (72.6%)	82 (27.4%)
Atherosclerosis is a type of cardiovascular disease	226 (75.1%)	74 (24.9%)
Do you think stress influences the progress of coronary heart disease? ]	261 (87.1%)	39 (12.9%)
Stress may cause an increase in blood sugar, blood pressure, and cholesterol levels	243 (81.1%)	57 (18.9%)
Rheumatic heart disease is a type of cardiovascular diseases	242 (80.6%)	58 (19.4%)
Myocardial oxygen supply is dependent on coronary blood flow	212 (70.6%)	88 (29.4%)
The older a person is, the greater their risk of having coronary heart disease	269 (89.6%)	31 (10.4%)
High blood sugar makes the heart work harder	213 (71.1%)	87 (28.9%)
I would seek medical care urgently, even if chest pain was of intermittent character	243 (81.1%)	57 (18.9%)
If your “bad” cholesterol (LDL) is high, you are at risk for heart disease	243 (81.1%)	57 (18.9%)

## Discussion

Globally, cardiovascular diseases (CVDs), a significant group of illnesses affecting the heart and blood arteries, are the leading cause of death; this pattern is also seen in Egypt. Since many of the risk factors for CVD can be changed and controlled early on, prevention initiatives should concentrate on raising public understanding of where these risk factors originate. Deficient knowledge in this area may contribute to unfavorable attitudes and the adoption of unhealthy lifestyles.

The current study revealed that females had a significantly higher level of knowledge about cardiovascular diseases and their risk factors compared to males (*P* = 0.007). This finding aligns with the results of a study conducted in Turkey in 2021^20^. This difference in knowledge levels may be attributed to women generally being more proactive in seeking health information and utilizing healthcare services, which increases their exposure to educational materials about cardiovascular diseases. These could explain the higher level of knowledge observed among females in both this study and the referenced Turkish study. Furthermore, students from Faculties of Dentistry, Pharmacy and Physical Therapy were more knowledgeable about CVDs and their risk factors than those who were from other colleges like Applied sciences, Engineering and Arts. This association suggests that exposure to health-related education may be linked to higher CVD knowledge, indicating the potential value of targeted educational programs for students in non-health disciplines.

The current study’s findings showed a significant association between the year of study and knowledge level, with students’ awareness of cardiovascular disease (CVD) rising as their educational level increases. Increased exposure to education throughout time may be the cause of this trend. Similar findings were reported in a study from Turkey, which assessed college students’ knowledge of cardiovascular disease risk factors (CVDRFs) and found that senior students demonstrated higher levels of knowledge compared to their first-year counterparts^20^.

A significantly higher proportion of respondents correctly identified family history (75.1%), age (89.6%), blood pressure (87.6%), blood sugar (70.1), Obesity (85.6), Physical inactivity (85.1%) and smoking (90%) as a risk factors for heart disease, indicating a generally good level of awareness regarding these risk factors. However, the fact that nearly one-quarter (24.9%) of respondents answered incorrectly highlights a concerning knowledge gap. These findings suggest the importance of developing targeted educational initiatives to strengthen understanding of CVD risk factors within prevention efforts. These findings aligns with a 2021 study from India, which reported that nearly 80% of participants had knowledge about modifiable risk factors namely hypertension, smoking, hyperlipidemia, diabetes mellitus, obesity, sedentary lifestyle and diet. Conversely, the same study indicated that participants had limited knowledge (approximately 40%) regarding non-modifiable risk factors like age, and genetics^21^.

While the American Heart Association acknowledges that more research is needed to fully understand the role of stress in the development of heart disease^22^, our study found that a significant majority of participants (87.1%) believed stress to be a major contributing factor to cardiovascular disease (CVD). This perception is consistent with the findings of Satyjeet et al. (2020), who also identified stress as one of the most commonly reported causes of CVD among their study population^23^. Furthermore, our study assessed participants’ understanding of cardiovascular function and found that 70% correctly answered questions related to the impact of blood sugar on heart health and the importance of coronary blood flow in supplying oxygen to the heart muscle. However, misconceptions were prevalent regarding cholesterol types, as 54.2% of participants provided incorrect answers about HDL and LDL cholesterol. Comparable findings were reported in separate studies conducted in Pakistan and India, which also documented limited understanding of cardiovascular risk factors among participants^21,24^. In contrast, a 2020 study from Oakland University in the United States demonstrated comparatively higher knowledge levels, with 67% of students correctly identifying optimal blood cholesterol levels and their respective physiological functions^25^.

In the context of emergency cardiovascular scenarios, 81.1% of participants correctly identified the necessity of seeking immediate medical attention for intermittent chest pain, demonstrating awareness of its potential seriousness. Similarly, 70.1% recognized the vital importance of timely intervention in managing acute myocardial infarction (AMI). These findings indicate a good level of awareness regarding the urgency of prompt medical attention in cardiovascular emergencies.

## Strengths and limitations

This study’s primary strength lies in its relatively diverse sample, comprising 300 participants drawn from seven different colleges within a private university in Egypt. This heterogeneity allows for a more comprehensive understanding of cardiovascular health knowledge across various academic disciplines. Additionally, the use of a structured questionnaire ensured consistency in data collection and facilitated objective comparison of responses. However, several limitations should be noted. As a cross-sectional study, it captures data at a single point in time, which limits the ability to infer causality. Moreover, as the study employed a convenience sampling method, the results may not be fully representative of the target population, and this should be acknowledged as a limitation affecting the generalizability of the findings. The study was conducted within a single private university, which may not reflect the broader population of Egyptian university students, particularly those from public institutions or different socioeconomic backgrounds. Self-reported data may also introduce response bias, as participants might have provided socially desirable answers rather than reflecting their true knowledge or beliefs. Nonetheless, the use of a previously validated and anonymous questionnaire helped minimize the risk of socially desirable responses and misreporting. Recall bias was unlikely, as the questions assessed factual knowledge rather than past experiences. Additionally, since all questions in the online form were mandatory, no missing or incomplete data were present, ensuring completeness and consistency of responses.

The participants had almost 80% knowledge about risk factors for CVD and < 50% knowledge about the role of age, genetics, diet, and cholesterol in the pathogenesis of CVD. To tackle the CVD burden and bridge the knowledge gap, it is essential to develop educational interventions that facilitate risk prediction and a risk-stratification approach at both the individual and community levels. Implementing heart-healthy behaviors at an early age can help postpone CVD-related clinical events, lower the case fatality rate, and reduce healthcare costs.

## Conclusion

Within the limitations of the current study, students in applied sciences, engineering, and arts had much less awareness of cardiovascular disease (CVD) than those in dentistry and pharmacy. Furthermore, more than half of the participants had significant misconceptions about the roles of HDL and LDL cholesterol in CVD. These findings emphasize the importance of focused health education programs focusing on diet and cholesterol awareness to improve CVD prevention.

## Data Availability

The datasets used and/or analyzed during the current study are available from the corresponding author on reasonable request.
